# Secondary Primary Malignancy Risk among Patients with Esophageal Cancer in Taiwan: A Nationwide Population-Based Study

**DOI:** 10.1371/journal.pone.0116384

**Published:** 2015-01-30

**Authors:** San-Chi Chen, Chung-Jen Teng, Yu-Wen Hu, Chiu-Mei Yeh, Man-Hsin Hung, Li-Yu Hu, Fan-Chen Ku, Cheng-Hwai Tzeng, Tzeon-Jye Chiou, Tzeng-Ji Chen, Chia-Jen Liu

**Affiliations:** 1 Division of Hematology and Oncology, Department of Medicine, Taipei Veterans General Hospital, Taipei, Taiwan; 2 Division of Hematology and Oncology, Department of Medicine, Far Eastern Memorial Hospital, Taipei, Taiwan; 3 School of Medicine, National Yang-Ming University, Taipei, Taiwan; 4 Cancer Center, Taipei Veterans General Hospital, Taipei, Taiwan; 5 Department of Family Medicine, Taipei Veterans General Hospital, Taipei, Taiwan; 6 Department of Psychiatry, Kaohsiung Veterans General Hospital, Kaohsiung, Taiwan; 7 Division of Hematology and Oncology, Department of Medicine, Changhua Show-Chwan Memorial Hospital, Changhua, Taiwan; 8 Division of Transfusion Medicine, Department of Medicine, Taipei Veterans General Hospital, Taipei, Taiwan; 9 Institute of Public Health, National Yang-Ming University, Taipei, Taiwan; Queen Mary Hospital, HONG KONG

## Abstract

**Background:**

To evaluate the risk and sites of metachronous secondary primary malignancies (SPMs) among patients with esophageal cancer.

**Methods:**

Newly diagnosed esophageal cancer patients between 1997 and 2011 were recruited. To avoid surveillance bias, SPMs that developed within one year were excluded. Standardized incidence ratios (SIRs) of metachronous SPMs in these patients were calculated by comparing to the cancer incidence in the general population. Risk factors for SPM development, included age, sex, comorbidities and cancer-related treatments, were estimated by Cox proportional hazards models.

**Results:**

During the 15-year study period, 870 SPMs developed among 18,026 esophageal cancer patients, with a follow-up of 27,056 person-years. The SIR for all cancers was 3.53. The SIR of follow-up period ≥ 10 years was 3.56; 5–10 years, 3.14; and 1–5 years, 3.06. The cancer SIRs of head and neck (15.83), stomach (3.30), lung and mediastinum (2.10), kidney (2.24) and leukemia (2.72), were significantly increased. Multivariate analysis showed that age ≥ 60 years (hazard ratio [HR] 0.74), being male (HR 1.46) and liver cirrhosis (HR 1.46) were independent factors. According to the treatments, major surgery (HR 1.24) increased the risk, but chemotherapy was nearly significant.

**Conclusions:**

Patients with esophageal cancer were at increased risk of developing metachronous SPMs. The SIR remained high in follow-up > 10 years, so that close monitoring may be needed for early detection of SPM among these esophageal cancer patients.

## Introduction

Esophageal cancer, one of the most serious malignancies with poor survival, has a high incidence in Southern Africa and Eastern Asia [1]. Squamous cell carcinoma (SCC) is the most common histological form (more than 80%), and Taiwan, one of the most prevalent countries in Eastern Asia, is increasing in SCC prevalence [2]. In Western countries, epidemiology results indicated an increasing trend of adenocarcinoma of the lower third esophagus [3], probably due to a higher prevalence of the main risk factors—obesity and Barrett’s esophagus[4,5]. A higher global health spending is to be expected, drawing a growing worldwide concern.

With the improved radiation planning and delivery methods and the introduction of neoadjuvant chemoradiotherapy, the overall 5-year survival rate has increased to 40%[6]. The issue of secondary primary malignancies (SPMs) following a primary esophageal cancer emerged subsequently because of a longer survival-lifespan and more aggressive chemotherapy and radiotherapy.

Two large cohort studies have shown an increased standardized incidence ratio (SIR) of SPM in esophageal cancer patients[7,8], but a heterogenously histological constitution[9] and inconsistent trends of histological types in different geographic regions[10] are of concern. In addition, only a few studies focused on the relationship between treatment and SPM[11,12]. Up to the present, there has been no large-scale study to demonstrate the increase of metachronous SPMs in Asia. Hence, it is worthwhile executing a nationwide population-based study on this issue.

The Taiwan National Health Insurance Research Dataset (NHIRD) is a nationwide population dataset supplied for health research. All malignancies are registered according to a strict protocol in the NHIRD are suitable for the analysis of SPM. NHIRD includes not only patients’ age and sex, but also full information on comorbidities, and cancer-related therapies such as surgery, chemotherapy and radiotherapy.

The aim of this study is to explore the risk of metachronous SPM in esophageal cancer patients by comparing with the general population. In addition, we also investigate the effects of treatment and comorbidities, which may be potential predispositions toward developing SPM.

## Methods

### Data Sources

Taiwan’s National Health Insurance (NHI) system, which began in 1995, is an obligatory universal health insurance program that provides comprehensive medical care to all the residents of Taiwan, with a coverage rate of over 99%[13]. It supplies medical care for outpatient, inpatient, emergency, dental, and traditional Chinese medicine services, including prescription drugs.

This current study retrieved data from the NHIRD database, which is managed by the National Health Research Institute of Taiwan. Furthermore, the NHI database of catastrophic illness (Registry of Catastrophic Illness) provides information on patients with severe diseases and integrates several NHI databases, such as claims data, NHI enrollment files, and the registry for drug prescriptions. All the malignancies are considered catastrophic illnesses, and the certification of any malignancy requires tissue pathologic proof for peer review. All NHI patient information is encrypted. For this reason, the institutional review board of Taipei Veterans General Hospital has exempted our study from full reviewing (2013-10-002CE).

### Study Population

This nationwide population-based cohort study, from January 1, 1997 to December 31, 2011, retrieved newly diagnosed esophageal cancers (ICD-9-CM 150) from the Registry of Catastrophic Illness. Patients aged less than 20 years at diagnosis or with antecedent malignancies were excluded. These patients were followed until the occurrence of SPM, death, or dropout from the NHI program before the end of the year 2011. Information about comorbidities and esophageal cancer treatments such as major surgery (including total esophagectomy, esophagogastrectomy), radiation and chemotherapy were collected for further analysis.

### Statistical Analyses

The main dependent variable was the occurrence of SPM. We used SIRs to determine the risk of SPM in this study cohort. SIRs are defined as the observed number of cancer development divided by the expected number, which is calculated by multiplying the patient numbers of our study cohort by the cancer incidence in the general population at the corresponding group. Each group was stratified in accordance with sex, calendar year, and age in five-year intervals by the corresponding stratum-specific person-time accrued in the general population. We acquired cancer incidence data among the general population from the Taiwan National Cancer Registry. The 95% confidence intervals (CIs) of the SIRs were estimated based on the assumption of the observed numbers of cancers abiding by a Poisson probability distribution. In addition, we defined the SIRs for subgroups according to sex and age. To avoid surveillance bias, we performed a subgroup analysis stratified by the period of developing SPM. For the same reason, we excluded those SPMs that occurred within one year to calculate SIRs for different types of cancers. We performed a cumulative incidence analysis to estimate the cumulative risk of SPM. Risk factors for development of SPM among esophageal cancer patients were analyzed with the use of univariate and multivariate Cox proportional hazards models. Variables such as age, sex, comorbidities and cancer-related therapies were included in the model, and p < 0.1 in the univariate model was then put into a Cox multivariable model.

Data were obtained and computed using the Perl programming language (version 5.12.2; Perl Foundation, Walnut, CA). Microsoft’s SQL Server 2012 (Microsoft Corp., Redmond, WA), was used for data linkage, processing, and sampling. In this study, we used SAS 9.2 software (SAS Institute Inc., Cary, NC) and STATA statistical software (version 11.0; StataCorp, College Station, TX) for statistical analyses. A p value < 0.05 was defined as statistical significance.

## Results

### Characteristics of the Study Population

**Figure 1:**
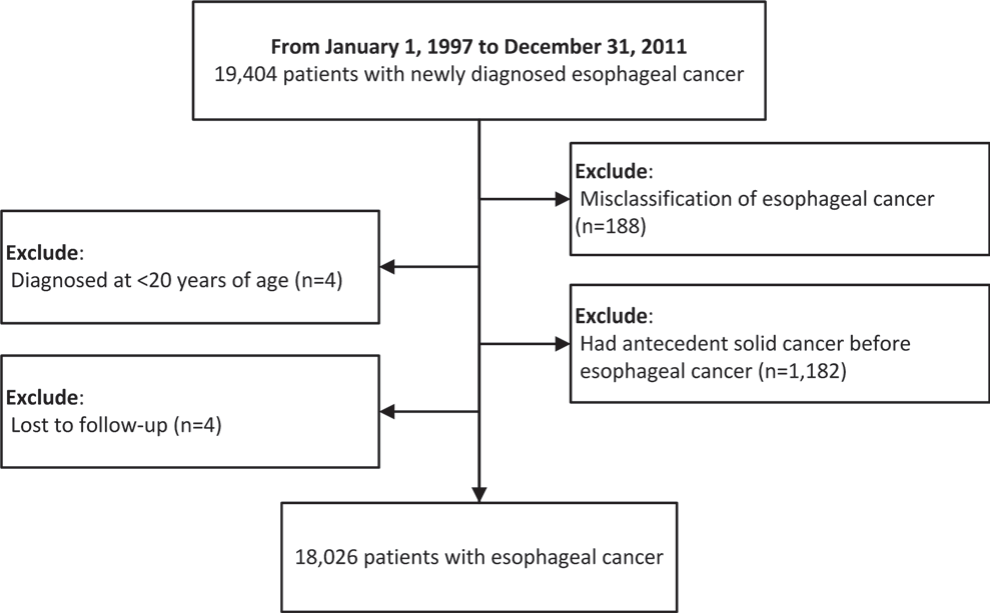
Flow-chart.

We identified 19,404 patients with esophageal cancer in the NHIRD catastrophic illness registry. Of them, 188 patients were misclassified, four patients were below 20 years of age, 1,182 patients had antecedent malignancies before esophageal cancer, and four patients were lost to follow-up after diagnosis (Fig. 1). Therefore, the final study cohort consisted of 18,026 patients, including 16,675 (92.5%) males and 1,351 (7.5%) females, with a median age of 59 years at diagnosis (interquartile range, 50–70 years) and a follow-up of 27,056 person-years. The characteristics of this cohort are shown in Table 1.

**Table 1:** Characteristics of patients with esophageal cancer.

	**Total**	**Male**	**Female**
No. of patients	18,026	16,675	1,351
Person-years at risk	27,056.4	24,393.4	2,663.04
Median follow-up, years (interquartile range)	0.76 (0.34–1.60)	0.75 (0.35–1.58)	0.86 (0.34–2.05)
Median age, years (interquartile range)	59 (50–70)	59 (50–69)	71 (59–80)
Age at diagnosis, years			
20–39	598	571	27
40–59	8,767	8,425	342
60–79	7,322	6,664	658
≥ 80	1,339	1,015	324

### All Cancers

During the observation period, 870 SPMs developed. To compare with the general population, patients with esophageal cancer had a significantly increased risk of overall SPMs (SIR 3.53, 95% CI 3.30–3.77, *p* < 0.001), both in men (SIR 3.63, 95% CI, 3.39–3.89, *p* < 0.001) and women (SIR 2.52, 95% CI, 1.90–3.28, *p* < 0.001). A subanalysis shows that patients aged 20–39 have a highest SIR of 36.56 (95% CI 23.42–54.40). Subgroup analysis based on the follow-up period showed significantly higher SIRs of period 0–1 (SIR 4.17, 95% CI 3.78–4.60, *p* < 0.001), 1–5 (SIR 3.06, 95% CI 2.74–3.42, *p* < 0.001), 5–10 (SIR 3.14, 95% CI 2.59–3.77, *p* < 0.001) and ≥10 years (SIR 3.56, 95% CI 2.26–5.34, *p* < 0.001). The results are shown in Table 2.

**Table 2:** Standardized incidence ratios according to sex, age at diagnosis and follow-up time of esophageal cancer.

**Characteristics**	**Total**			**Male**			**Female**		
	**Observed**	**Expected**	**SIR (95% CI)**	**Observed**	**Expected**	**SIR (95% CI)**	**Observed**	**Expected**	**SIR (95% CI)**
All cancers	870	246.31	3.53 (3.30–3.77)	815	224.49	3.63 (3.39–3.89)	55	21.82	2.52 (1.90–3.28)
Age at diagnosis, years									
20–39	24	0.66	36.56 (23.42–54.40)	23	0.58	39.33 (24.93–59.01)	1	0.07	13.96 (0.35–77.79)
40–59	446	50.47	8.84 (8.04–9.70)	425	47.59	8.93 (8.10–9.82)	21	2.88	7.30 (4.52–11.16)
60–79	348	157.68	2.21 (1.98–2.45)	324	144.70	2.24 (2.00–2.50)	24	12.98	1.85 (1.18–2.75)
≥ 80	52	37.51	1.39 (1.04–1.82)	43	31.62	1.36 (0.98–1.83)	9	5.89	1.53 (0.70–2.90)
Follow-up time after esophageal cancer									
0–1	412	98.69	4.17 (3.78–4.60)	380	91.22	4.17 (3.76–4.61)	32	7.47	4.29 (2.93–6.05)
1–5	320	104.51	3.06 (2.74–3.42)	304	95.31	3.19 (2.84–3.57)	16	9.20	1.74 (0.99–2.82)
5–10	115	36.66	3.14 (2.59–3.77)	109	32.50	3.35 (2.75–4.05)	6	4.16	1.44 (0.53–3.14)
≥ 10	23	6.46	3.56 (2.26–5.34)	22	5.46	4.03 (2.52–6.10)	1	1.00	1.00 (0.03–5.59)

SIR, standardized incidence ratio; CI, confidence interval

### Specific Cancer Types

**Figure 2:**
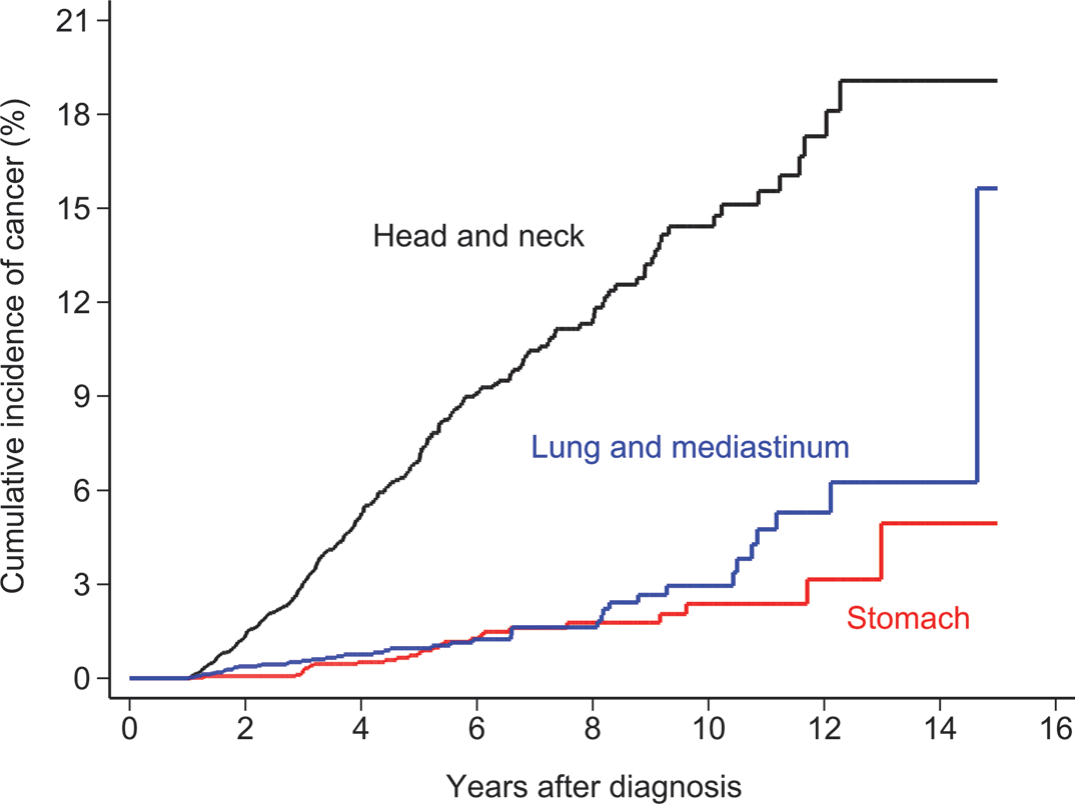
Cumulative incidence plot of selective cancers after esophageal cancer.

To focus on the metachronous SPMs, we excluded those SPMs developed within one year following diagnosis of esophageal cancer. SIRs were significantly higher in cancers of the head and neck (15.83, 95% CI 13.94–17.90), stomach (3.30, 95% CI 2.24–4.69), lung and mediastinum (2.10, 95% CI 1.56–2.77), kidneys (2.24, 95% CI 1.03–4.26), as well as in leukemia (2.72, 95% CI 1.00–5.92). SIRs for specific types of cancers are shown in detail in Table 3. (Analysis including those SPMs developed within one year is shown in S1 Table) With a follow-up period of 5–10 years, increased SIRs were observed among cancers of the head and neck (17.25, 95% CI 13.10–22.29), lung and mediastinum (1.96, 95% CI 1.01–3.42), and stomach (4.76, 95% CI 2.38–8.51). With a follow-up time of >10 years, the SIRs remained significantly high in cancers of the head and neck (16.61, 95% CI 7.17–32.73), lung and mediastinum (6.37, 95% CI 2.56–13.12). SIRs for specific cancer types by different follow-up periods are displayed in Table 4, and the cumulative incidence plot of SPMs of head and neck, stomach, lung and mediastinum are showed in Fig. 2.

**Table 3:** Standardized incidence ratios (SIRs) for specific cancer types among patients with esophageal cancer (follow-up more than 1 year).

**Site of cancers**	**Total**			**Male**			**Female**		
	**Observed**	**Expected**	**SIR (95% CI)**	**Observed**	**Expected**	**SIR (95% CI)**	**Observed**	**Expected**	**SIR (95% CI)**
All cancers	458	147.62	3.10 (2.82–3.40)	435	133.27	3.26 (2.96–3.59)	23	14.35	1.60 (1.02–2.40)
Head and neck	253	15.98	15.83 (13.94–17.90)	248	15.62	15.87 (13.96–17.98)	5	0.36	13.91 (4.52–32.46)
Digestive	94	64.03	1.47 (1.19–1.80)	88	58.12	1.51 (1.21–1.87)	6	5.91	1.01 (0.37–2.21)
Stomach	31	9.39	3.30 (2.24–4.69)	29	8.61	3.37 (2.26–4.84)	2	0.79	2.55 (0.31–9.20)
Colon and rectum, anus	24	24.97	0.96 (0.62–1.43)	20	22.29	0.90 (0.55–1.39)	4	2.68	1.49 (0.41–3.82)
Liver and biliary tract	32	26.62	1.20 (0.82–1.70)	32	24.53	1.30 (0.89–1.84)	0	2.09	0.00 (0.00–1.77)
Pancreas	7	3.05	2.29 (0.92–4.72)	7	2.69	2.60 (1.04–5.35)	0	0.36	0.00 (0.00–10.25)
Lung and mediastinum	50	23.83	2.10 (1.56–2.77)	45	22.01	2.04 (1.49–2.74)	5	1.82	2.74 (0.89–6.40)
Bone and Soft tissue	4	1.13	3.53 (0.96–9.03)	3	1.03	2.90 (0.60–8.49)	1	0.10	9.87 (0.25–54.99)
Skin	7	3.11	2.25 (0.90–4.63)	6	2.65	2.26 (0.83–4.92)	1	0.46	2.16 (0.05–12.04)
Breasts	2	2.23	0.90 (0.11–3.24)	0	0.15	0.00 (0.00–24.32)	2	2.08	0.96 (0.12–3.48)
Genitourinary	35	27.20	1.29 (0.90–1.79)	32	24.81	1.29 (0.88–1.82)	3	2.39	1.25 (0.26–3.66)
Cervix	1	0.84	1.19 (0.03–6.62)	N/A	N/A	N/A	1	0.84	1.19 (0.03–6.62)
Uterus	0	0.30	0.00 (0.00–12.10)	N/A	N/A	N/A	0	0.30	0.00 (0.00–12.10)
Ovaries	1	0.27	3.70 (0.09–20.62)	N/A	N/A	N/A	1	0.27	3.70 (0.09–20.62)
Prostate	16	15.00	1.07 (0.61–1.73)	16	15.00	1.07 (0.61–1.73)	N/A	N/A	N/A
Bladder	8	6.78	1.18 (0.51–2.32)	8	6.34	1.26 (0.55–2.49)	0	0.44	0.00 (0.00–8.32)
Kidneys	9	4.01	2.24 (1.03–4.26)	8	3.47	2.30 (0.99–4.54)	1	0.53	1.87 (0.05–10.42)
Thyroid	0	1.05	0.00 (0.00–3.50)	0	0.78	0.00 (0.00–4.73)	0	0.27	0.00 (0.00–13.48)
Hematologic	9	6.64	1.35 (0.62–2.57)	9	5.99	1.50 (0.69–2.85)	0	0.65	0.00 (0.00–5.68)
Non-Hodgkin’s lymphoma	3	3.31	0.91 (0.19–2.65)	3	2.97	1.01 (0.21–2.95)	0	0.34	0.00 (0.00–10.92)
Hodgkin’s disease	0	0.12	0.00 (0.00–30.90)	0	0.11	0.00 (0.00–32.84)	0	0.01	0.00 (0.00–523.36)
Multiple myeloma	0	1.01	0.00 (0.00–3.65)	0	0.91	0.00 (0.00–4.06)	0	0.10	0.00 (0.00–36.09)
Leukemia	6	2.21	2.72 (1.00–5.92)	6	2.00	2.99 (1.10–6.52)	0	0.20	0.00 (0.00–18.17)
All others	4	2.40	1.67 (0.45–4.27)	4	2.10	1.91 (0.52–4.88)	0	0.30	0.00 (0.00–12.40)

SIR, standardized incidence ratio; CI, confidence interval; N/A, not applicable

**Table 4:** Numbers of cases and SIRs of specific cancer types by follow-up time.

**Site of cancers**	**1–5 y**		**5–10 y**		**≥ 10 y**	
	**Observed**	**SIR (95% CI)**	**Observed**	**SIR (95% CI)**	**Observed**	**SIR (95% CI)**
All cancers	376	2.64 (2.38–2.92)	179	2.40 (2.06–2.78)	40	3.04 (2.17–4.14)
Head and neck	187	15.40 (13.28–17.78)	58	17.25 (13.10–22.29)	8	16.61 (7.17–32.73)
Digestive	62	1.36 (1.04–1.74)	27	1.72 (1.13–2.50)	5	1.84 (0.60–4.29)
Stomach	18	2.69 (1.60–4.26)	11	4.76 (2.38–8.51)	2	5.01 (0.61–18.10)
Colon and rectum, anus	18	1.03 (0.61–1.63)	5	0.79 (0.26–1.84)	1	0.87 (0.02–4.82)
Liver and biliary tract	21	1.09 (0.67–1.66)	9	1.43 (0.65–2.71)	2	1.95 (0.24–7.06)
Pancreas	5	2.34 (0.76–5.46)	2	2.58 (0.31–9.32)	0	0.00 (0.00–25.93)
Lung and mediastinum	31	1.87 (1.27–2.65)	12	1.96 (1.01–3.42)	7	6.37 (2.56–13.12)
Bone and soft tissue	3	3.70 (0.76–10.82)	1	3.63 (0.09–20.23)	0	0.00 (0.00–76.25)
Skin	4	1.90 (0.52–4.87)	2	2.38 (0.29–8.62)	1	5.84 (0.15–32.53)
Breasts	2	1.37 (0.17–4.94)	0	0.00 (0.00–5.89)	0	0.00 (0.00–26.57)
Genitourinary	22	1.18 (0.74–1.79)	11	1.52 (0.76–2.72)	2	1.46 (0.18–5.29)
Cervix	1	1.67 (0.04–9.31)	0	0.00 (0.00–18.02)	0	0.00 (0.00–95.49)
Uterus	0	0.00 (0.00–18.49)	0	0.00 (0.00–42.56)	0	0.00 (0.00–197.24)
Ovaries	1	5.62 (0.14–31.32)	0	0.00 (0.00–48.10)	0	0.00 (0.00–236.70)
Prostate	8	0.79 (0.34–1.56)	7	1.70 (0.68–3.50)	1	1.26 (0.03–7.00)
Bladder	6	1.27 (0.47–2.76)	2	1.15 (0.14–4.16)	0	0.00 (0.00–11.76)
Kidneys	6	1.27 (0.47–2.76)	2	1.98 (0.24–7.17)	1	5.46 (0.14–30.41)
Thyroid	0	0.00 (0.00–4.78)	0	0.00 (0.00–15.39)	0	0.00 (0.00–89.07)
Hematologic	6	1.29 (0.47–2.80)	3	1.79 (0.37–5.22)	0	0.00 (0.00–12.20)
Non-Hodgkin’s lymphoma	1	0.43 (0.01–2.40)	2	2.39 (0.29–8.63)	0	0.00 (0.00–24.62)
Hodgkin’s disease	0	0.00 (0.00–42.05)	0	0.00 (0.00–135.43)	0	0.00 (0.00–836.01)
Multiple myeloma	0	0.00 (0.00–5.24)	0	0.00 (0.00–14.32)	0	0.00 (0.00–77.30)
Leukemia	5	3.23 (1.05–7.53)	1	1.80 (0.05–10.01)	0	0.00 (0.00–36.73)
All others	3	1.76 (0.36–5.15)	1	1.82 (0.05–10.13)	0	0.00 (0.00–40.81)

SIR, standardized incidence ratio; CI, confidence interval; N/A, not applicable

### Predictive Factors for Secondary Primary Malignancies

We calculated the cumulative incidence and hazard ratio (HR) with a time-dependent covariate in the Cox regression model. Univariate Cox proportional hazards analysis showed that age ≥ 60 years, being male, and having cirrhosis were significantly associated with cancer development. Multivariate analysis showed that age ≥ 60 years (HR 0.74, 95% CI 0.64–0.85, *p* < 0.001), being male (HR 1.46, 95% CI 1.11–1.93, *p* = 0.007), and being diagnosed with cirrhosis (HR 1.46, 95% CI 1.21–1.76, *p* < 0.001) remained independent predictors of SPM development. In the analysis of treatments of esophageal cancer, major surgery (HR 1.24, 95% CI 1.06–1.44, *p* = 0.006) was the independent risk factor of subsequent SPM; chemotherapy and radiotherapy were not. These results are shown in Table 5.

**Table 5:** Risk factors for cancer development in patients with esophageal cancer (N = 18,026).

**Variables**	**Univariate analysis**		**Multivariate analysis^a^**	
	**HR (95% CI)**	***P* Value**	**HR (95% CI)**	***P* Value**
Age ≥ 60	0.67 (0.59–0.77)	<0.001	0.74 (0.64–0.85)	<0.001
Sex (male)	1.64 (1.25–2.16)	<0.001	1.46 (1.11–1.93)	0.007
**Comorbidities**				
Diabetes mellitus	1.12 (0.94–1.32)	0.198		
COPD	0.95 (0.81–1.10)	0.471		
Liver cirrhosis	1.58 (1.32–1.90)	<0.001	1.46 (1.21–1.76)	<0.001
Autoimmune diseases	0.90 (0.65–1.26)	0.545		
Dyslipidemia	1.05 (0.88–1.26)	0.575		
ESRD	1.08 (0.73–1.59)	0.707		
**Treatment^b^**				
Major surgery	1.27 (1.10–1.47)	0.002	1.24 (1.06–1.44)	0.006
Chemotherapy	1.14 (0.99–1.32)	0.060	1.14 (0.99–1.32)	0.076
Radiotherapy	0.91 (0.79–1.05)	0.207		

Abbreviations: COPD, chronic obstructive pulmonary disease; ESRD, end-stage renal disease

^a^All factors with *p*< 0.1 in univariate analyses were included in the Cox multivariate analysis.

^b^Treatment was analyzed as a time-dependent covariate in the Cox regression model.

## Discussion

This is the largest nationwide population-based cohort study in Asia to determine SPM risk among esophageal cancer patients. Our major findings include: (1) the risk of metachronous SPM was significantly increased, even after ten years of follow-up; (2) the SPMs with a significantly increased SIR included cancers of the head and neck, stomach, lung and mediastinum, kidneys, and leukemia; (3) the independent risk factors for SPM were being male, having cirrhosis and having undergone major surgery.

The SPM risk following a primary esophageal cancer has been seldom reported in large-scale studies. Chuang et al. reported an increased risk (SIR 1.34) of SPM among esophageal cancer patients in 13 population-based cancer registries of different countries[7]. Zhu et al. also reported a similar result (SIR 1.34) by the use of the SEER dataset, though with unknown histological types[8]. In Asia, Matsubara et al. presented the SIR of 2.98 in esophageal squamous cell carcinoma patients in Japan[14]. That the SIR in the Japan cohort was more than double of the non-Asia cohort might be attributed to the difference of the histological spectrum in different geographic regions. However, a small number of subjects (only 679 patients), coupled with a single institute experience, may limit the power of study analysis in the Japan cohort. By using the NHIRD in Taiwan, this large cohort study, showing an SIR of 3.53 serves as a more convincing and supportive report on the Asian region. In addition, with the advantage of comprehensive follow-up, our study provides a significantly higher SIR in an even longer follow-up period (that means, > 10 years) than studies by Chung[7] and Zhu[8] et al., in which the follow-up was limited to 5–10 years.

The most common sites of SPMs associated with esophageal cancer are the aerodigestive tract organs, such as the head and neck, lungs, and the stomach,[7,11,14,15] which was explained by the carcinogenic effects of tobacco and alcohol on the other parts of the aerodigestive tract simultaneously[16]. Our study supports this concept, and we found that the SIRs were significantly high in cancers of the head and neck, lung and mediastinum, even with follow-up of >10 years. Accompanied with Fig. 2 that shows the increasing cumulative incidence plot of these cancers with long-term follow-up, it is suggest that the effect of field cancerization might persist for a long time.

It has been reported that head and neck cancer is the most common SPM after a primary esophageal cancer. The SIRs are especially high in Japan and in our population[14], relative to those in Western studies[7,8]. This may be due to the different risk factors between the East and West. In Asia, squamous cell carcinoma is the major type of esophageal cancer and it is associated with alcohol intake and tobacco use. [17] Head and neck cancer also shares the same risk factor.[18,19]

Regarding to the subsequent lung cancer, it may be difficult to absolutely exclude the possibility of pulmonary metastasis of esophageal cancer. But with the exclusion of those lung cancer developed within one year and the excess of risk persisted even at ten more years, we consider the chance of bias minimal.

Stomach cancer is also a common SPM. Chuang et al. demonstrated that esophageal adenocarcinoma has a higher SIR of gastric cancer than esophageal squamous cell carcinoma[7]. Nevertheless, esophageal squamous cell carcinoma still shows a significantly increased SIR of stomach cancer in other studies[8,14]. Besides the sharing of common risk factors between esophageal and gastric cancers, another explanation is that gastric cancer risk increases following gastrectomy in esophageal cancer patients[20].

The SIRs of kidney cancer[7] and leukemia[21] were also significantly increased, as observed in previous studies. The common risk factor between kidney and esophageal cancers is smoking[22]. On the other hand, secondary leukemia may be related to chemotherapy and radiotherapy[23]. However, the case numbers of both cancers are small, so it is possible that the associations are due to chance.

In the multivariate analysis, our study demonstrated that cirrhosis was an independent risk factor of SPM. Very little data has shown an increased cancer risk in patients with cirrhosis[24]. In addition, diabetes reportedly leads to an increased risk of cancer[25]. However, diabetes was not found to be a risk factor for SPM in the esophageal cancer patients that make up our cohort.

Our study also shows that major surgery (HR 1.24) increases the risk of SPM, and chemotherapy (HR 1.14, 95% CI 0.91–1.32) is almost significant. Those patients who received surgery were younger and had earlier stages. They were expected to live longer and might have higher chances to develop SPMs. On the other hand, doctors tend to perform more intensive disease investigation in post-operation patients if they are suspected to have a new lesion, thus more SPMs were discovered.

Generally, chemotherapy and radiotherapy are considered to have cytotoxic effects on other organs and thus increase sequential cancer risk. Our study reveals that chemotherapy is only nearly significant, while radiotherapy is not. The results were controversial in previous studies. Matsubara et al. presented that both treatments were not correlated with SPM[14], but Zhu et al. stated that these treatments may increase risk[8].

This retrospective study has several limitations. First, we did not know the histological types of esophagus cancers. The risk factors of each type are not the same and those factors may be associated with SPMs. However, squamous cell carcinoma accounts for about 95% of all esophageal cancers in Taiwan[26], so it’s fair to assume that our cohort is similar in this respect. Second, the higher incidence of SPMs may relate to close surveillance or misclassification. It is well known that esophageal cancer, head and neck cancer, and lung cancer often developed synchronously[27]. Additionally, recurrent metastatic tumors in the lungs may be misclassified as primary lung squamous cell carcinoma. Hence, we excluded those SPMs diagnosed within one year. In addition, the cumulative incidence of SPM was increased after five years. The result was less likely related to misclassification and close surveillance. Lastly, several potential confounders, including obesity, tobacco and alcohol use, genetic alteration and family malignancy history could not be analyzed.

In conclusion, we demonstrate that the risk of metachronous SPM is significantly increased among esophageal cancer patients. With follow-ups lasting more than 10 years, the SIR was still high. Therefore, after the first standard five years of surveillance, longer monitoring may be considered for early detection of SPM, especially cancers of head and neck, lung and mediastinum.

## Supporting Information

S1 Table Standardized incidence ratios (SIRs) for specific cancer types among patients with esophageal cancer. SIR, standardized incidence ratio; CI, confidence interval; N/A, not applicable
(DOC)
