# Supplementary material for: Secondary Primary Malignancy Risk among Patients with Esophageal Cancer in Taiwan: A Nationwide Population-Based Study
Source: PLoS One. 2015 Jan 30;10(1):e0116384. doi: 10.1371/journal.pone.0116384 (PMC4312084; doi:10.1371/journal.pone.0116384)
Supplement: S1 Table — SIR, standardized incidence ratio; CI, confidence interval; N/A, not applicable (DOC) [file pone.0116384.s001.doc]

**S1 Table Standardized incidence ratios (SIRs) for specific cancer types among patients with e**sophageal cancer

|  | Total |  |  |  | Male |  |  |  | Female |  |  |
| --- | --- | --- | --- | --- | --- | --- | --- | --- | --- | --- | --- |
| Site of cancers | Observed | Expected | SIR (95% CI) |  | Observed | Expected | SIR (95% CI) |  | Observed | Expected | SIR (95% CI) |
| All cancers | 870 | 246.31 | 3.53 (3.30–3.77) |  | 815 | 224.49 | 3.63 (3.39–3.89) |  | 55 | 21.82 | 2.52 (1.90–3.28) |
| Head and neck | 432 | 28.69 | 15.06 (13.67–16.54) |  | 417 | 28.14 | 14.82 (13.43–16.31) |  | 15 | 0.55 | 27.16 (15.20–44.79) |
| Digestive | 238 | 107.41 | 2.22 (1.94–2.52) |  | 219 | 98.42 | 2.23 (1.94–2.54) |  | 19 | 8.99 | 2.11 (1.27–3.30) |
| Stomach | 113 | 15.94 | 7.09 (5.84–8.52) |  | 103 | 14.71 | 7.00 (5.71–8.49) |  | 10 | 1.23 | 8.11 (3.89–14.92) |
| Colon and rectum, anus | 47 | 41.14 | 1.14 (0.84–1.52) |  | 41 | 37.05 | 1.11 (0.79–1.50) |  | 6 | 4.09 | 1.47 (0.54–3.20) |
| Liver and biliary tract | 71 | 45.28 | 1.57 (1.22–1.98) |  | 68 | 42.16 | 1.61 (1.25–2.04) |  | 3 | 3.12 | 0.96 (0.20–2.81) |
| Pancreas | 7 | 5.04 | 1.39 (0.56–2.86) |  | 7 | 4.49 | 1.56 (0.63–3.21) |  | 0 | 0.54 | 0.00 (0.00–6.77) |
| Lung and mediastinum | 102 | 39.20 | 2.60 (2.12–3.16) |  | 94 | 36.45 | 2.58 (2.08–3.16) |  | 8 | 2.75 | 2.90 (1.25–5.72) |
| Bone and soft tissue | 10 | 1.92 | 5.22 (2.50–9.60) |  | 8 | 1.76 | 4.54 (1.96–8.95) |  | 2 | 0.15 | 12.92 (1.57–46.68) |
| Skin | 10 | 5.03 | 1.99 (0.95–3.66) |  | 9 | 4.33 | 2.08 (0.95–3.94) |  | 1 | 0.70 | 1.43 (0.04–7.99) |
| Breasts | 3 | 3.36 | 0.89 (0.18–2.61) |  | 0 | 0.26 | 0.00 (0.00–14.21) |  | 3 | 3.10 | 0.97 (0.20–2.83) |
| Genitourinary | 47 | 43.76 | 1.07 (0.79–1.43) |  | 42 | 40.03 | 1.05 (0.76–1.42) |  | 5 | 3.72 | 1.34 (0.44–3.13) |
| Cervix | 2 | 1.39 | 1.44 (0.17–5.21) |  | N/A | N/A | N/A |  | 2 | 1.39 | 1.44 (0.17–5.21) |
| Uterus | 0 | 0.46 | 0.00 (0.00–8.09) |  | N/A | N/A | N/A |  | 0 | 0.46 | 0.00 (0.00–8.09) |
| Ovaries | 2 | 0.41 | 4.86 (0.59–17.54) |  | N/A | N/A | N/A |  | 2 | 0.41 | 4.86 (0.59–17.54) |
| Prostate | 19 | 23.66 | 0.80 (0.48–1.25) |  | 19 | 23.66 | 0.80 (0.48–1.25) |  | N/A | N/A | N/A |
| Bladder | 9 | 11.22 | 0.80 (0.37–1.52) |  | 9 | 10.55 | 0.85 (0.39–1.62) |  | 0 | 0.67 | 0.00 (0.00–5.50) |
| Kidneys | 15 | 6.62 | 2.27 (1.27–3.74) |  | 14 | 5.82 | 2.40 (1.31–4.03) |  | 1 | 0.80 | 1.25 (0.03–6.98) |
| Thyroid | 8 | 1.81 | 4.43 (1.91–8.73) |  | 7 | 1.39 | 5.03 (2.02–10.37) |  | 1 | 0.41 | 2.41 (0.06–13.44) |
| Hematologic | 15 | 11.02 | 1.36 (0.76–2.24) |  | 14 | 10.04 | 1.39 (0.76–2.34) |  | 1 | 0.98 | 1.02 (0.03–5.70) |
| Non-Hodgkin's lymphoma | 7 | 5.49 | 1.28 (0.51–2.63) |  | 6 | 4.98 | 1.20 (0.44–2.62) |  | 1 | 0.51 | 1.98 (0.05–11.01) |
| Hodgkin's disease | 0 | 0.21 | 0.00 (0.00–17.98) |  | 0 | 0.19 | 0.00 (0.00–18.97) |  | 0 | 0.01 | 0.00 (0.00–342.09) |
| Multiple myeloma | 0 | 1.65 | 0.00(0.00–2.23) |  | 0 | 1.50 | 0.00 (0.00–2.45) |  | 0 | 0.15 | 0.00 (0.00–24.29) |
| Leukemia | 8 | 3.67 | 2.18 (0.94–4.29) |  | 8 | 3.37 | 2.38 (1.03–4.68) |  | 0 | 0.31 | 0.00 (0.00–11.99) |
| All others | 5 | 4.12 | 1.21 (0.39–2.83) |  | 5 | 3.66 | 1.37 (0.44–3.19) |  | 0 | 0.46 | 0.00 (0.00–8.04) |

SIR, standardized incidence ratio; CI, confidence interval; N/A, not applicable
